# Are patients with preoperative synovitis suitable for unicompartmental knee arthroplasty? Magnetic resonance imaging evidence from a retrospective cohort study

**DOI:** 10.1186/s12891-023-06506-7

**Published:** 2023-05-30

**Authors:** Zhengxi Wang, Xudong Zhang, Xianzuo Zhang, Jiaxing Wang, Chen Zhu

**Affiliations:** 1 Wannan Medical College, Wuhu, China; 2 Department of Orthopedics, The Affiliated Provincial Hospital of Anhui Medical University, Anhui Medical University, Hefei, China; 3 Division of Life Sciences and Medicine, The First Affiliated Hospital of USTC, University of Science and Technology of China, Hefei, China; 4 Department of Orthopedics Shanghai Sixth People’s Hospital Affiliated to Shanghai Jiao Tong University School of Medicine, Shanghai Jiao Tong University, Shanghai, China

**Keywords:** Synovitis, Knee joints, Knee osteoarthritis, Unicompartmental knee arthroplasty, Cartilage loss

## Abstract

**Background:**

The use of unicompartmental knee arthroplasty (UKA) in patients with preoperative synovitis is controversial. This study aimed to investigate the association between synovitis detected by magnetic resonance imaging (MRI) and prognosis after UKA.

**Methods:**

Synovitis was graded using the MRI Osteoarthritis Knee Score criteria based on preoperative MRI findings of 132 UKAs performed between June 2020 and August 2021. The Knee Society Knee Score (KS-KS) and the Knee Society Function Score were collected preoperatively and 1 year postoperatively. The relationship between synovitis and the changes in the Knee Society score was analyzed using logistic regression.

**Results:**

Univariate logistic regression showed that patients with higher preoperative synovitis scores (odds ratio (OR) = 1.925, 95% confidence interval (CI): 1.482–2.500, *P* < 0.001) had higher KS-KS changes. After adjusting for confounding variables, synovitis was proven to be an independent factor for KS-KS improvement after UKA in multivariate logistic regression (OR = 1.814, 95% CI: 1.354–2.430, *P* < 0.001). Before UKA, patients with synovitis had lower pain scores (PS) than patients without synovitis (95% CI: -17.159 – -11.160, t = -9.347, *P* < 0.001). There was no difference in PS between the two groups after UKA (95% CI: -6.559 – 0.345, t = -1.782, *P* = 0.077).

**Conclusions:**

Patients with synovitis can achieve good improvement of pain symptoms, and the efficacy is not inferior to that of non-synovitis patients after UKA.

**Supplementary Information:**

The online version contains supplementary material available at 10.1186/s12891-023-06506-7.

## Background

Knee osteoarthritis (KOA) is a degenerative joint disease with joint failure caused by a combination of factors such as advanced age, abnormal cartilage metabolism, and abnormal biomechanics [1]. The main features of KOA are cartilage loss and synovitis, directly related to clinical symptoms, such as joint swelling and inflammatory pain [2]. Studies have shown that in the United States, approximately 500,000 people each year require joint replacement due to irreversible KOA progression. Furthermore, the quality of life of these patients is impaired, increasing the social healthcare burden [3, 4].

For patients with isolated medial KOA, surgical treatment options include unicompartmental knee arthroplasty (UKA), total knee arthroplasty (TKA), and high tibial osteotomy [5–8]. Previous cohort studies have shown that UKA has a 10-year survival rate of 90%. With the application of minimally invasive surgical techniques [8] and the improvement of implant design [9], UKA has been widely used to treat KOA. More than 70% of patients with KOA experience single-compartment degeneration at a particular stage of the disease. UKA during this period can effectively intervene and restore joint stability to achieve the purpose of minimally invasive surgeries.

The indications for UKA are mainly anteromedial KOA and radiological examination, showing the “bone-to-bone” (full-thickness cartilage loss) of the medial compartment in the anteroposterior weight-bearing position [10]. With the popularization of magnetic resonance imaging (MRI) examination technology, traditional radiological evaluation of the indications for UKA surgery has been effectively supplemented [11, 12]. MRI can help obtain more information on soft tissue lesions of the knee joint and perform corresponding semi-quantitative [13, 14] and quantitative [15] analyses of injuries, such as meniscus injury, cruciate ligament injury, partial-thickness cartilage loss (CL), bone marrow lesions (BMLs), and synovitis. However, the findings of previous studies that have explored the relationship between related soft tissue lesions and UKA postoperative outcomes are controversial [16, 17]. In addition, there are few studies on the efficacy of UKA in patients with synovitis. Studies have shown that CL may cause pain through an indirect pathway primarily mediated by worsening synovitis rather than bone marrow damage [18]. Therefore, synovitis is closely related to KOA pain symptoms.

Pain relief and restoration of knee function are the main goals of UKA. Therefore, this study aimed to investigate the postoperative efficacy of UKA in patients with KOA with synovitis and its relationship with pain relief. We speculated that patients with anteromedial KOA with synovitis are unsuitable for UKA and that their postoperative pain symptoms would not be significantly improved.

## Methods

### Study population

**Fig. 1:**
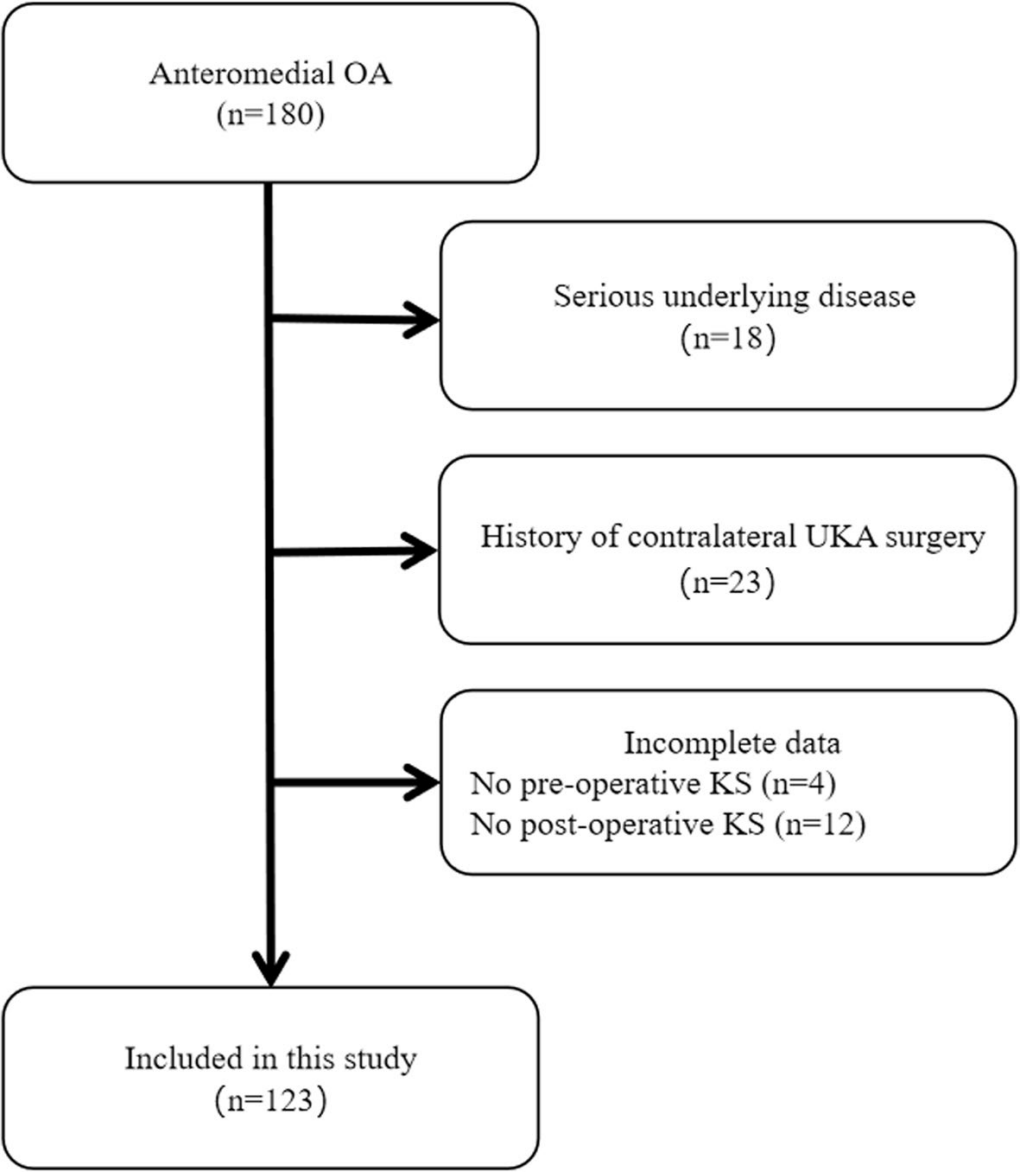
Study design and flowchart

This study was conducted in accordance with the principles of the Declaration of Helsinki and was approved by the ethics committee. The medical records of patients hospitalized in the Department of Orthopedics of the First Affiliated Hospital of the University of Science and Technology of China and who underwent UKA between June 2020 and August 2021 were selected for this retrospective study. Of the 180 patients initially considered, 123 knees were included in the study (Fig. 1). The inclusion criteria for the study were as follows: (1) ability to obtain preoperative MRI data of the knee joint; (2) having no apparent cognitive impairment and ability to cooperate with the research investigation; and (3) follow-up period of at least 1 year. The exclusion criteria were as follows: (1) severe underlying diseases and lower extremity nerve injury or surgical history; (2) patients who received bilateral UKA treatment at the same time; and (3) patients with incomplete medical records or missing follow-up information. Overall, the data of 123 eligible patients were collected. A high-volume joint replacement specialist performed the above 123 UKA procedures using a single-implant design (Medial Mobile-Bearing UKA). A total of 123 patients were followed up postoperatively for at least 1 year.

Moreover, a total of 123 knees underwent knee MRI at baseline, and some structural features were assessed semi-quantitatively. Evaluations included synovitis, BMLs, CL, and injuries to the anterior cruciate ligament (ACL), medial meniscus (MM), and lateral meniscus (LM). The assessors included an attending physician specializing in joint surgery and another from the imaging department. Disputes were jointly decided by a third party after consultation.

### BMLs and CL

We used the MRI Osteoarthritis Knee Score (MOAKS) [14] for semi-quantitative measures of the BMLs and CL. In the MOAKS, the knee joint was divided into 14 zones for scoring the CL and BMLs (a subspinous area was added for scoring the BMLs). The tibia was divided into three medial (anterior, medial, and posterior) and three lateral (anterior, medial, and posterior) subregions, covered by articular cartilage and a subspinous subregion. The femur was divided into six subregions: medial and lateral trochlear, medial and lateral femur central, and posteromedial and lateral femur. The patella in the axial plane was divided into two subregions: medial and lateral. The BMLs were graded from 0 to 3 according to the lesion volume: 0 indicated no lesion, 1 indicated that the lesion fills 1/3 of the bony area, 2 indicated that the lesion fills 1/3 to 2/3 of the bony area, and 3 indicated that the lesion fills 2/3 of the bony area; CL was described similarly. The main body of this study included patients undergoing medial UKA. According to the classification method by Jacobs et al. [19], the BML evaluation mainly assesses the medial compartment, including the medial tibia, medial femur, and patella, with a total of eight subregions (score range of each region is 0–3); the overall medial-BML score is 0–24. The CL includes the medial tibia and femur with six subregions (scores ranging from 0 to 3 for each region and 0 to 18 for the total medial-CL score).

### Synovitis

The MOAKS study used two new terms, "Hoffa-synovitis" and "Effusion-synovitis" [14]. Hoffa-synovitis refers to hyperintensity within the Hoffa fat pad and serves as a surrogate marker for synovitis on non-contrast-enhanced MRI. Effusion-synovitis is synonymous with "joint effusion," a term indicating that MRI-detected joint effusion constitutes inflamed synovial membrane and fluid. Therefore, we created a synovitis summary score (range 0–6) using the sum of Hoffa-synovitis (range 0–3, score is based on size: 0 = normal, 1 = mild, 2 = moderate, and 3 = severe) and Effusion-synovitis scores (0 = physiologic amount, 1 = small: fluid continuous in the retropatellar space, 2 = medium: with slight convexity of the suprapatellar bursa, and 3 = large: evidence of capsular distention).

### Meniscus

Meniscus MRI diagnostic classification [20]: grade I is the focal abnormal signal in the meniscus; grade II is the linear high signal reaching the articular surface in the meniscus; and grade III is the linear high signal reaching the articular surface. Grades I and II represent meniscus deformation; Grade III represents tearing.

#### ACL

ACL features, such as intact and degenerative changes and completely torn on MRI, were noted [21, 22]. According to previous studies [23, 24], intact and degenerative (functionally intact: > 14% posterior intact cartilage of the medial compartment) features were classified as the ACL-functional group, while completely torn and degenerative (functionally insufficient: < 14% posterior intact cartilage of the medial compartment) features were regarded as the ACL-deficient (ACLD) group.

### Knee Society score(KSS)

The KSS includes the Knee Society Knee Score (KS-KS) and the Knee Society Function Score (KS-FS) subsets [25], each of which ranges from 0 to 100, with higher scores indicating better results. The KS-KS was scored partly by the assessors and partly reported by the patients. The assessors measured range of motion (ROM), and stability (0–50). Participant self-reported pain level (0–50). The KS-KS was further subdivided into pain score (PS)、ROM score and stability score. The KS-FS was self-reported based on the walking distance (0–50) and the ability to climb and descend stairs (0–50). Deductions were made based on the use of the walker. According to Lee et al. [26], the minimal clinically significant difference identified for the KS-FS is between 6.1 (95% confidence interval (CI): 5.1–7.1) and 6.4 (95% CI: 4.4–8.4) and between 5.3 (95% CI: 4.3–6.3) and 5.9 (95% CI 3.9–7.8) for KS-KS. Therefore, a "good" outcome was defined as a change in the KS-KS ≥ 38.67 and a change in the KS-FS ≥ 16.48 after 1 year of follow-up.

### Statistical analyses

Univariate and multivariate logistic regression models were established to analyze the predictors of preoperative MRI findings and prognosis after UKA. The magnitude of this association is expressed as odds ratio (OR) and CI. A -value of *P* < 0.05 indicates a statistically significant difference. All analyses were performed using SPSS Statistics V25 (SPSS, Armonk, NY, USA).

## Results

### Patient baseline characteristics

The characteristics of the study participants are shown in Table 1. The participants were mainly women (78.86%), with a mean age of 63 (standard deviation (SD) = 8.58) years and a mean body mass index of 26.2 (SD = 3.49). The mean follow-up was 1.5 years (SD = 0.22). Among the 123 cases with preoperative knee MRI findings, the semi-quantitative data on BMLs and synovitis had a skewed distribution. The proportion of ACLD was 23.6%, and MM had the highest rate of third-degree injury (62.6%). The proportion of hypertension among the underlying diseases was higher (33.3%).

**Table 1:** Patient characteristics (*n* = 123)

	Mean/Median/Ratio
Age (years)	63 (8.58)
Female sex, % (No.)	78.86% (97)
BMI (kg/m^2^)	26.2 (3.49)
Follow-up (y)	1.5 (0.22)
BML score (MOAKS, 0–24)	4 (2, 7)
CL score (MOAKS, 0–18)	9.46 (2.832)
Synovitis score (MOAKS, 0–6)	2 (0, 3)
ACLD, % (No.)	23.6% (29)
MM, % (No.)	
0	1.6% (2)
I	7.3% (9)
II	28.5% (35)
II	62.6% (77)
LM, % (No.)	
0	11.4% (14)
I	32.5% (40)
II	39.0% (48)
III	17.1% (21)
Self-reported disease, % (No.)	
Normal	56.1% (69)
Hypertension	33.3% (41)
Diabetes	2.4% (3)
Cardiovascular disease	5.7% (7)
Heart disease	5.7% (7)
Pre KS-KS (KSS, 0–100)	46.3 (11.87)
Pre KS-FS (KSS, 0–100)	59.9 (13.74)

*BMI* Body mass index, *BML* Bone marrow lesion, *CL* Cartilage loss, *MOAKS* MRI Osteoarthritis Knee Score, *ACLD* Anterior cruciate ligament deficient, *MM* Medial meniscus, *LM* Lateral meniscus, *KSS* Knee Society scores, *KS-KS* Knee Society Knee Scores, *KS-FS* Knee Society Function Scores

### The change trend of each score pre-and postoperative

**Fig. 2:**
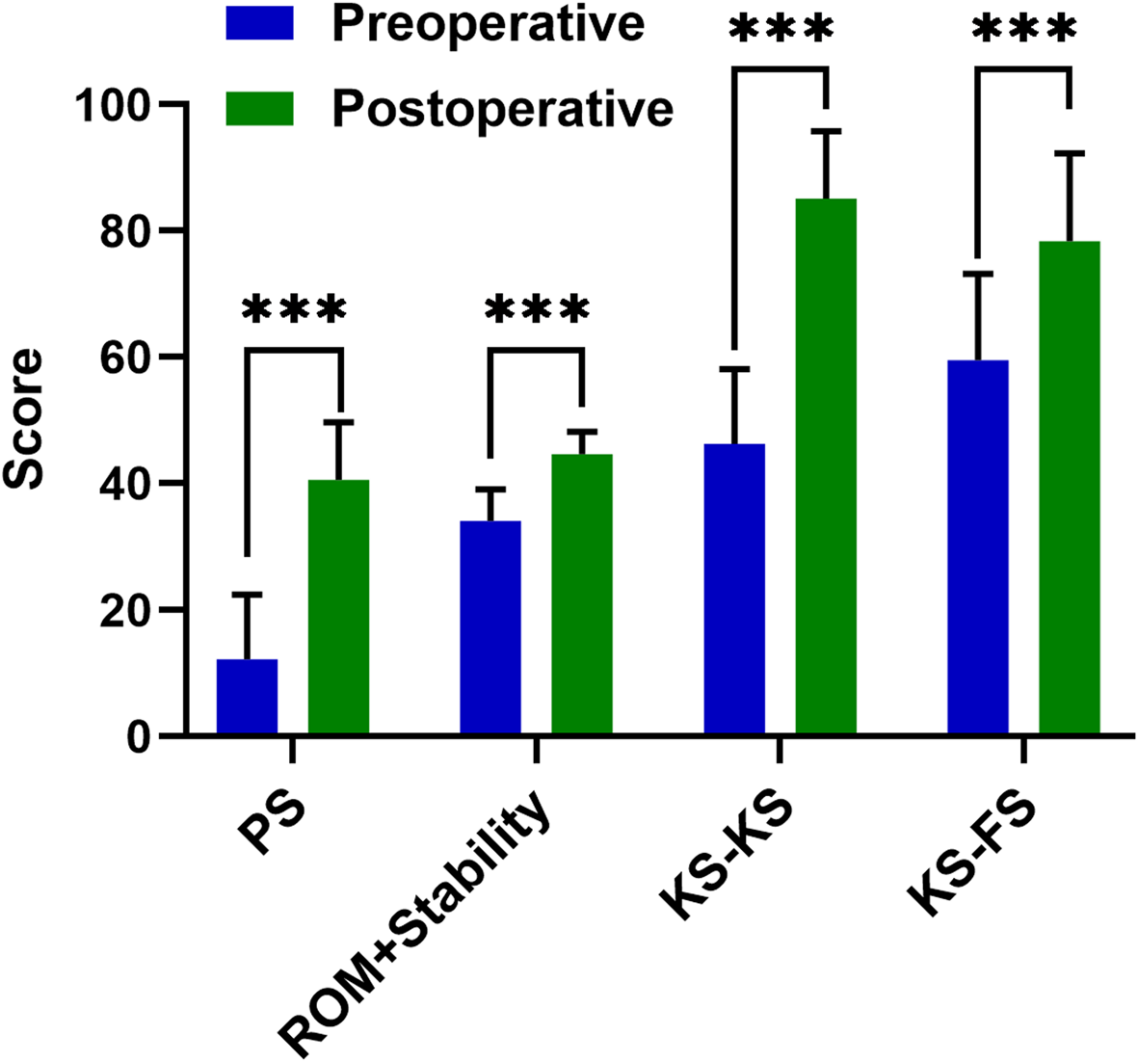
The change trend of each score before and after operation. The T test was used for intra-group differences. The values are shown as mean ± SD (*n* = 123) and *** indicates *P* < 0.001. *PS *Pain scores, *ROM *Range of motion, *KS-KS* Knee Society Knee Scores, *KS-FS* Knee Society Function Scores.

We analyzed changes in each item of the KSS assessment. The results indicated that KS-KS change had the most significant impact. Prior to the operation, KS-KS was at 46.25 ± 11.869 and increased to 85.16 ± 10.607 after the operation (*P* < 0.001). This increase was mainly caused by changes in the PS score, which improved from 12.20 ± 10.286 to 40.53 ± 9.139 (*P* < 0.001), followed by improvements in ROM and stability score from 34.06 ± 5.017 to 44.63 ± 3.560 (*P* < 0.001) (Fig. 2).

### Univariate analyses of the changes in the KS-KS predictors

The univariate logistic regression analysis findings for predicting the changes in the KS-KS are shown in Table 2. Synovitis (3 vs. 0, *P* < 0.001) and CL (10.45 vs. 8.34, *P* < 0.001) were closely related to the prognosis of UKA. The specific performance was that the synovial inflammation and CL were more severe, and the changes in the KS-KS were "good."

**Table 2:** Predictors of changes in the KS-KS — univariate analyses

	Changes in the KS-KS			If *P*-value < 0.05	
	Good (*n* = 65)	Poor (*n* = 58)	*P*-value^a^	OR	95% CI
Female sex, % (No.)	73.8% (48)	84.5% (49)	0.153		
Age (years), mean (SD)	62.3 (9.09)	63.9 (7.95)	0.287		
BMI (kg/m^2^), mean (SD)	26.3 (3.47)	26.1 (3.53)	0.777		
BML score	4 (2,7)	4 (1,6)	0.186		
CL score	10.45(2.87)	8.34(2.36)	< 0.001	1.377	1.168–1.623
Synovitis score	3 (2,4)	0 (0,2)	< 0.001	1.925	1.482–2.500
ACLD, % (No.)	24.6% (16)	22.4% (13)	0.774		
MM, % (No.)					
0	0	3.4% (2)	0.188		
I	7.7% (5)	6.9% (4)			
II	24.6% (16)	32.8% (19)			
III	67.7% (44)	56.9% (33)			
LM, % (No.)					
0	9.2% (6)	13.8% (8)	0.974		
I	35.4% (23)	29.3% (17)			
II	40.0% (26)	37.9% (22)			
III	15.4% (10)	19.0% (11)			

^a^Logistic regression to predict good outcome indicators of changes in the KS-KS

*KS-KS* Knee Society Knee Scores, *BMI* Body mass index, *BML* Bone marrow lesion, *CL* Cartilage loss, *ACLD* Anterior cruciate ligament deficient, *MM* Medial meniscus, *LM* Lateral meniscus, *OR* Odds ratio, *CI* Confidence interval

### Univariate analyses of the changes in the KS-FS predictors

The univariate logistic regression analysis findings for predicting the changes in the KS-FS are shown in Table 3. Although no significant independent variables were obtained, the effect of BMLs (5 vs. 3, *P* = 0.069) on the changes in the KS-FS scores showed a positive trend.

**Table 3:** Predictors of changes in the KS-FS — univariate analyses

	Changes in the KS-FS			If *P*-value < 0.05	
	Good (*n* = 72)	Poor(*n* = 51)	*P*-value^a^	OR	95% CI
Female sex, % (No.)	75.0% (54)	84.3% (43)	0.216		
Age (years), mean (SD)	63.2 (8.79)	62.8 (8.35)	0.826		
BMI (kg/m^2^), mean (SD)	26.3 (3.40)	26.0 (3.63)	0.711		
BML score	5 (2,7)	3 (0,6)	0.069		
CL score	9.63 (2.84)	9.22 (2.84)	0.429		
Synovitis score	2 (0,4)	2 (0,3)	0.350		
ACLD, % (No.)	26.4% (19)	19.6% (10)	0.384		
MM, % (No.)					
0	1.4% (1)	2.0% (1)	0.522		
I	9.7% (7)	3.9% (2)			
II	27.8% (20)	29.4% (15)			
III	61.1% (44)	64.7% (33)			
LM, % (No.)					
0	12.5% (9)	9.8% (5)	0.762		
I	30.5% (22)	35.3% (18)			
II	41.7% (30)	35.3% (18)			
III	15.3% (11)	19.6% (10)			

^a^Logistic regression to predict good outcome indicators of changes in the KS-FS

*KS-FS* Knee Society Function scores, *BMI*, Body mass index, *BML* Bone marrow lesion, *CL* Cartilage loss, *ACLD* Anterior cruciate ligament deficient, *MM* Medial meniscus, *LM* Lateral meniscus, *OR* Odds ratio, *CI* Confidence interval

### Multivariate analyses of good outcome predictors of UKA

The multivariate logistic regression model used to predict the outcome after UKA is shown in Table 4. CL, BMLs, ACLD, MM, LM, and synovitis were included to construct a multivariate logistic regression equation. The results showed that severe synovitis (OR = 1.812, 95% CI: 1.360–2.414, *P* < 0.001) and CL (OR = 1.245, 95% CI: 1.039–1.490, *P* = 0.017) were associated with better improvement in the KS-KS changes, with a statistically significant difference.

**Table 4:** Predictors of good outcome indicators of UKA — multivariate analyses

	OR	95% CI	*P*-value^a^
Changes in the KS-KS			
BML score	1.006	0.895–1.131	0.925
CL score	1.245	1.039–1.490	0.017
Synovitis score	1.812	1.360–2.414	< 0.001
ACLD	1.170	0.425–3.220	0.761
MM	1.372	0.720–2.615	0.336
LM	0.658	0.402–1.078	0.097
Changes in the KS-FS			
BML score	1.094	0.985–1.214	0.093
CL score	1.023	0.886–1.182	0.753
Synovitis score	1.079	0.857–1.360	0.516
ACLD	1.530	0.631–3.710	0.347
MM	0.799	0.459–1.388	0.425
LM	0.922	0.599–1.421	0.714

^a^Multivariate logistic regression to predict good outcome indicators of UKA

*KS-KS* Knee Society Knee scores, *KS-FS* Knee Society Function scores, *BML* Bone marrow lesion, *CL* Cartilage loss, *ACLD* Anterior cruciate ligament deficient, *MM* Medial meniscus, *LM* Lateral meniscus, *OR* Odds ratio, *CI* Confidence interval

Table 5 shows the logistic regression analysis of synovitis before and after adjusting for covariates. Overall, synovitis and changes in the KS-KS were significantly associated (OR = 1.925, 95% CI: 1.482–2.500, *P* < 0.001). Furthermore, after adjusting for age, sex, body mass index, BML, ACLD, MM, LM, and CL, the association remained significant (OR = 1.814, 95% CI: 1.354–2.430, *P* < 0.001).

**Table 5:** Logistic regression analysis of synovitis before and after adjusting for the covariates

	Odds ratio	95% confidence interval	*P*-value
Crude	1.925	1.482–2.500	< 0.001
Model 1	1.958	1.495–2.564	< 0.001
Model 2	1.987	1.496–2.639	< 0.001
Model 3	1.814	1.354–2.430	< 0.001

Crude univariate logistic regression analysis: Synovitis was included as an independent variable, and changes in the KS-KS was included as a dependent variable.

Model 1: This model included synovitis and demographic factors as covariates for the independent variable.

Model 2: In addition to those included in Model 1, bone marrow lesion, anterior cruciate ligament, medial meniscus, and lateral meniscus were added as independent variables.

Model 3: In addition to those included in Model 2, cartilage was added as an independent variable.

### Association of synovitis with pre-and postoperative PS

Table 6 shows that the preoperative PS was 7.59 ± 7.585 in the synovitis group and 21.75 ± 8.439 in the non-synovitis group. There was a significant difference in the mean score between the two groups (95% CI: -17.159 – -11.160, t = -9.347, *P* < 0.001). There was no significant difference in postoperative PS between the two groups (95% CI: -6.559 – 0.345, t = -1.782, *P* = 0.077).

**Table 6:** Comparison of pre-and postoperative PS between groups with and without synovitis

	Synovitis (*n* = 83)	Non-synovitis (*n* = 40)	95%CI	T-text	
				T-value	*P*-value^a^
Per PS	7.59 ± 7.585	21.75 ± 8.439	-17.159 – -11.160	-9.347	< 0.001
Post PS	39.52 ± 10.049	42.63 ± 6.503	-6.559 – 0.345	-1.782	0.077

^a^*P*-value represents the assessment for group differences

*PS* Pain scores, *CI* Confidence interval

### Short-term Postoperative Complications

Table 7 shows that postoperative venous thrombosis accounted for 14.5% in the synovitis group and 10% in the non-synovitis group. There was no significant difference in distribution between groups (*P* = 0.687). Similarly, there was no significant difference in the distribution of delayed wound healing between the two groups (7.2% vs 10%, *P* = 0.861).

**Table 7:** Distribution of short-term complications between groups with and without synovitis

Complication	Synovitis (*n* = 83)	Non-synovitis (*n* = 40)	Chi-square test	
			Chi-square value	*P*‑value^a^
Venous thrombosis				
(16/123)	12(14.5%)	4(10%)	0.162	0.687
Delayed wound healing				
(10/123)	6(7.2%)	4(10%)	0.030	0.861

^a^*P*-value represents the assessment for group differences

## Discussion

This study hypothesized that patients with synovitis are not candidates for UKA. However, the results showed that the postoperative PS of patients with and without synovitis are similar. There was also no difference in the incidence of short-term postoperative complications between the two groups. Therefore, the postoperative outcome of patients with synovitis was not inferior to that of patients without synovitis. We then discuss the possible causes of pain improvement in patients with synovitis and the clinical value of MRI in patients with UKA.

Some studies have used the change in synovial tissue volume as an indicator of the KOA analgesia test. In this study of 120 patients with KOA who received intra-articular steroid injections, subsequent contrast-enhanced MRI reduction in synovial volume was associated with improved knee pain [29]. Therefore, the pain symptoms of KOA are closely related to the synovial volume and are mainly caused by synovial inflammation and the release of biological mediators. Surgical treatment can effectively remove the diseased synovial tissue, achieving the expected clinical effect. Although there are few studies on the relationship between the degree of synovitis and the outcome of UKA, previous studies have shown that KOA patients with synovitis have a significant effect after TKA. Su et al. [27] performed synovectomy and total knee replacement in 28 patients with synovitis. Short-term efficacy assessments were satisfactory, with mean KS-KS improving from 38.9 ± 9.5 (range: 17–54) to 84.4 ± 6.1 (range: 75–98). Matthew et al. [28] conducted a long-term efficacy study of 48 patients with synovitis after TKA with a mean follow-up time of 14 years. The results showed that the 10-year disease-free survival rate was 88%, and mean KS-KS and KS-FS were significantly improved postoperatively (*P* < 0.001). Although UKA has a smaller surgical incision than TKA, resecting the diseased synovial tissue is not ideal and residual synovial tissue may lead to arthritic pain. This is also one of the reasons why we speculated that UKA has poor efficacy. However, patients with KOA with severe synovial lesions before UKA had more severe pain symptoms. Therefore, postoperative pain symptoms were significantly relieved in these patients, and the surgical satisfaction was higher than that of patients with mild or no synovitis.

Articular cartilage has no intrinsic vascular or lymphatic supply; hence, it relies on adjacent tissues for support, such as the subchondral bone and synovium [30]. The synovium contains highly metabolically active synovial cells that nourish chondrocytes through the synovial fluid and joint space and remove metabolites and products of matrix degradation [31]. Notably, the synovium is essential for maintaining normal cartilage. However, inflamed synovium can produce catabolic and proinflammatory mediators, such as cytokines, nitric oxide, prostaglandin E2, and neuropeptides, and alter the balance of cartilage matrix degradation and repair, leading to the overproduction of cartilage-degrading proteolytic enzymes. The release of molecules from the degraded hyaline cartilage into the synovial cavity may amplify synovial inflammation in KOA, forming a vicious cycle [31]; hence, synovitis is inseparable from cartilage damage. The UKA corrected the deviation of the lower limb alignment caused by the cartilage defect and removed the diseased synovial tissue. Breaking the vicious cycle of catabolic and proinflammatory mediator interactions between synovitis and cartilage damage may be one of the reasons for the marked improvement in postoperative pain symptoms in severe synovitis.

The use of MRI before UKA surgery is a topic of debate in the medical community. MRI can accurately identify knee joint cartilage wear, synovium inflammation, meniscus injury, anterior and posterior cruciate ligament, lateral collateral ligament and other soft tissue lesions [12]. MRI can also rule out inflammatory arthropathy and diagnose osteonecrosis early [11]. One study calculated the sensitivity and specificity of MRI for the diagnosis of KOA to be 61% and 82%, respectively [32]. Therefore, MRI has certain advantages for the preoperative imaging evaluation of UKA patients. However, the classical indications of UKA are mainly symptoms and signs combined with radiological diagnosis, and MRI is not included [33]. More studies have also shown that MRI abnormalities do not necessarily affect the outcome of UKA surgery, and routine use of MRI may not be necessary. For example, Hurst et al. [34] study compared the postoperative KSS and failure rate between the MRI abnormal group (*n* = 33) and the rest of the patients (*n* = 967). There was no difference in survival or clinical outcome between the two groups. Therefore, MRI has a limited role in the assessment of arthritis and in the preoperative planning or decision making of UKA. In addition, from the perspective of cost-effectiveness analysis. In a study of 145 patients with moderate to severe KOA, 19 (13.1%) presented with an MRI scan. Physicians (*P* = 0.018) and academic groups (*P* = 0.044) ordered fewer MRIs than non-physicians and non-academic groups [35]. Therefore, most orthopedic surgeons prefer that patients with radiologically significant OA do not need MRI. The same conclusion was reached in the present study: abnormal MRI findings (synovitis) did not affect the UKA outcome. Routine use of MRI may even mislead the surgeon to make incorrect surgical decisions. However, MRI can be useful in identifying certain conditions that may affect the success of UKA surgery, such as the presence of infection or other underlying conditions. Ultimately, the decision whether to use MRI before UKA surgery should be made on a case-by-case basis, taking into account the individual patient's medical history and the potential benefits and risks of using MRI.

Our study has certain limitations which should be noted. Firstly, the retrospective nature of this study introduces a potential selection bias. Secondly, the sample size was not sufficient enough to allow for more detailed subgroup analyses. Thirdly, the follow-up time was too short to observe any long-term complications related to UKA. This lack of long-term observation limits the ability to assess postoperative outcomes in a comprehensive manner. Finally, Patient Reported Outcome Measures (PROMs) data were not collected, which limits our ability to evaluate the patient's subjective feelings and satisfaction postoperatively. Despite these limitations, this study is still important because, to the best of our knowledge, previous studies have not focused on the efficacy of synovitis on the outcome after UKA. Future studies should aim to investigate the impact of synovitis on long-term postoperative outcomes.

## Conclusion

Patients with synovitis can achieve good improvement of pain symptoms, and the efficacy is not inferior to that of non-synovitis patients after UKA.

## Supplementary Information

**Additional file 1.**

## Data Availability

All data generated or analyzed during this study are included in this published article and its supplementary information files.

## Abbreviations

KOA: Knee osteoarthritis
UKA: Unicompartmental knee arthroplasty
TKA: Total knee arthroplasty
MRI: Magnetic resonance imaging
KSS: Knee Society Score
KS-KS: Knee Society Knee Score
KS-FS: Knee Society Function Score
PS: Pain Score
ROM: Range of motion
CL: Cartilage loss
BMLs: Bone marrow lesions
ACL: Anterior cruciate ligament
MM: Medial meniscus
LM: Lateral meniscus
